# Antioxidant and anti-inflammatory activities of *Centratherum anthelminticum* (L.) Kuntze seed oil in diabetic nephropathy via modulation of Nrf-2/HO-1 and NF-κB pathway

**DOI:** 10.1186/s12906-022-03776-x

**Published:** 2022-11-18

**Authors:** Nida Baig, Rabia Sultan, Shamim Akhtar Qureshi

**Affiliations:** 1 Clinical Laboratory Sciences, Institute of Medical Technology, Dow University of Health Sciences, OJHA Campus, Karachi, Pakistan; 2 Department of Biochemistry, University of Karachi, Karachi, Pakistan; 3 Dr. Panjwani Center for Molecular Medicine and Drug Research, International Center for Chemical and Biological Sciences (ICCBS), University of Karachi, 75270, Karachi, Pakistan

**Keywords:** *Centratherum anthelminticum*, Fixed oil, Diabetes mellitus, Nephropathy, Nrf-2/Keap1/HO-1, NF-κB

## Abstract

**Background:**

Type 2 diabetes mellitus (T2DM) approximately constitutes 90% of the reported cases. 30-40% of diabetics eventually develop diabetic nephropathy (DN); accounting for one of the major causes of morbidity and mortality. Increased glucose autoxidation and non-enzymatic glycation of proteins in diabetic kidneys lead to the excessive generation of reactive oxygen species (ROS) that results in lipid peroxidation and activation of inflammatory mediators which overwhelms the scavenging capacity of the antioxidant defense system (Nrf2/Keap1/HO-1). *Centratherum anthelminticum* commonly called as kali zeeri (bitter cumin) and its seeds are well known for culinary purposes in Asia (Pakistan). It has reported anti-inflammatory, antioxidant, and anti-diabetic activities. The present study has attempted to explore the in-vivo anti-inflammatory, antioxidant and antihyperglycemic potential of the *C. anthelminticum* seed’s fixed oil (FO) and its fractions in high fat-high fructose-streptozotocin (HF-HFr-STZ) induced T2DM rat model.

**Methods:**

The T2DM rat model was developed by giving a high-fat and high-fructose diet followed by a single intraperitoneal injection of streptozotocin (STZ 60 mg/kg) on 28th day of the trial. After 72 hours of this injection, rats showing fasting blood glucose (FBG) levels≥230 mg/dL were recruited into six groups. These groups were orally administered distilled water (1 mL/kg), Gliclazide (200 mg/kg), *Centratherum anthelminticum* seed (FO) and its hexane (HF), chloroform (CF) and ethanol (EF) soluble fractions (200 mg/kg each), respectively for 4 weeks (i.e. 28 days). Blood, serum, and kidney tissue samples of euthanized animals were used for biochemical, pro-inflammatory, and antioxidant markers (ELISA, qRT-PCR, and spectrophotometric assays) and histology, respectively.

**Results:**

*C. anthelminticum* FO and its fractions reduced the lipid peroxidation, and improved the antioxidant parameters: enzymatic (SOD, CAT, and GPx), non-enzymatic (GSH), and mRNA expression of anti-inflammatory markers (Nrf-2, keap1, and HO-1). mRNA expression of inflammatory and apoptotic markers (TNF-α, IL-1β, COX-1, NF-**κ**B, Bax, and Bcl-2) were attenuated along with improved kidney architecture.

**Conclusion:**

*C. anthelminticum* can mitigate inflammation and oxidative stress in early DN. The anti-nephropathic effect can be attributed to its ability to down-regulate NF-κB and by bringing the Nrf-2 expression levels to near normal.

**Supplementary Information:**

The online version contains supplementary material available at 10.1186/s12906-022-03776-x.

## Introduction

The prevalence and incidence of diabetes mellitus (DM) are steadily increasing globally. According to the International Diabetes Federation (IDF), the prevalence of this multifactorial metabolic pandemic will increase to 10.2% (578 million) and 10.9% (700 million) by the year 2030 and 2045, respectively [1]. It is exerting a heavy toll on both the individual and society in the form of associated microvascular and macrovascular complications, especially diabetic nephropathy (DN). 30-40% of diabetics eventually develop DN, one of the most common and major causes of morbidity and mortality that eventually causes end-stage renal disease (ESRD) requiring either hemodialysis or renal transplant [2, 3].

Complex and multifactorial pathogenesis of DN is attributed to persistent hyperglycemia due to insufficient secretion or action of endogenous insulin [4]. The imbalance between secretion and action of insulin leads to increased glucose autoxidation, non-enzymatic glycation of proteins, and lipid peroxidation. Damaged biomolecules trigger excess generation of reactive oxygen species (ROS) that overwhelms the scavenging capacity of antioxidant defense systems: Nrf-2/Keap1/HO-1. This reflects not only in the form of cellular damage by hampering the endoplasmic reticulum (ER) and mitochondrial function rather also simultaneously triggering pro-inflammatory nuclear factor-kappa B (NF-κB), apoptotic (Bcl-2 and Bax), and pro-oxidant signaling cascade [5–9]. Hence, these cellular perturbations exert significant abnormalities on renal structure (podocytes, mesangial and tubular cells) which phenotypically presents as increased urinary albumin excretion, decreased glomerular filtration rate (GFR), and increased peripheral arterial blood pressure with subsequent ESRD [10–13]. Currently employed anti-glycemic, anti-hypertensive and management modalities have failed to slow the progression of DN and improve the patient’s survival due to limited efficacy and side effects. This calls for holistic treatment and management approaches more towards natural and medicinal plant-derived products with a focus on both cellular and molecular switches/signaling pathways that are involved in the pathogenesis of DN. The outlook mentioned above has the capacity to address metabolic, oxidative, and inflammatory insults which lie at the crux of pathogenesis; with the additive benefit of being relatively safe, with fewer side effects, and available at low cost. Moreover, several studies support the hypothesis that phyto-molecules with potent antioxidant and anti-inflammatory activities can delay and/or halt the progression of DN [14, 15].

Twenty-six plants for the management of Diabetes mellitus have been identified in folk medicine from Rayalaseema [16]. *Centratherum anthelminticum* (synonym: *Vernonia anthelmintica*, plant name corresponds to the latest revision mentioned in www.theplantlist.org and http://mpns.kew.org), an annual, erect, robust herb is one of them. It is commonly known as kalijiri, bitter cumin or Purple Fleabane. Seeds of this herb are most widely used to treat skin conditions, gastrointestinal problems, diabetes, fever, pulmonary fibrosis, and in the removal of worms and parasites, etc. [17]. It is also widely used as an ingredient in polyherbal formulations (PF) from India (Krumighattini, Rasaganthi Mezhugu, Perukala rasayanam and Kayakalp) [18–22], Sri Lanka (Navratri Kalka) [23] and China [24, 25].. It has been used in the traditional system of medicine to treat diabetes [26]. Sabu MC and Bhatia et al. had claimed that oral administration of the aqueous extract (100, 200, and 500 mg/kg, respectively) of seeds for more than 7 days in alloxan-induced diabetic rats significantly decreased (39%) serum glucose levels [27, 28]. However, Bhatia et al. found no significant change in glucose levels at higher doses; and he also found that polyphenolic enriched fraction of seed (50–200 mg/kg) containing quercetin, kaempferol, caffeic acid, gallic acid, proto-catechuic acid, ellagic acid, and ferulic acid exerted antidiabetic effect by inhibiting α-amylases and intestinal α-glucosidases in rat models [29]. Similarly, in 2010 Fatima et al., using streptozotocin-induced diabetic rat models showed significant antidiabetic (decreased levels in plasma glucose, HbA1c, plasma insulin, and hepatic glycogen) and antihyperlipidemic (decreased levels in cholesterol, triglycerides, LDL, VLDL, HDL, free fatty acids, phospholipids, and HMG-CoA reductase) activity of ethanolic extract and bioassay-directed fractions of *C. anthelminticum* (20 mg/kg) as compared to glibenclamide [30]. Antihyperlipidemic, antiatherogenic and antioxidant activities of ethanolic and crude extract of the seeds were also explored in high-fat diet-induced hyperlipidemic animal models with increasing doses from 200 to 600 mg/kg [31]. Carrageenan, cotton pellet and Freund’s adjuvant-induced paw edema, granuloma and arthritis in rats were the inflammatory models used by Otari et al. group to explore the effect of *C. anthelminticum* on inflammation [32]. The seed extracts also inhibited increasing levels of nitric oxide and inflammatory markers like IL-1β, IL-6, and TNF-α. Furthermore, the antioxidant potential explored by various groups in different time frames have shown that seeds extracted with methanol and ethanol and leaves extracted with hexane, chloroform, acetone, and methanol have free radical scavenging activity evident by DPPH and FRAP assays [19, 27, 33]. No in-vivo study exists in which the effect of *C. anthelminticum* on the innate antioxidant defense mechanisms has been explored at molecular level. Similarly, in the literature, only one ethno-medicinal study has been documented in which the oil from the seed of *C. anthelminticum* has been employed to treat skin disorders [34]. Therefore, the present study has attempted to explore the in-vivo antidiabetic, anti-inflammatory, antioxidant and anti-apoptotic potential of fixed oil (FO) extracted from *C. anthelminticum* and its hexane (HF), chloroform (CF), and ethanol (EF) in high fat-high-fructose-streptozotocin (HF-HFr-STZ)-induced T2DM rat model. Special attention was given to explore the nephroprotective effect of *C. anthelminticum* at the molecular/cellular level using a high fat-high-fructose-streptozotocin (HF-HFr-STZ)-induced T2DM rat model. Hence, the aim of the present study was to examine the effect of *C. anthelminticum* seed oil in mitigating the risk of end-organ complications observed in DM.

## Materials and methods

### Plant material

Seeds of *C. anthelminticum* were purchased from Hamdard Dawakhana, Saddar, Karachi. Identification was confirmed by the experts from the Department of Botany, University of Karachi, Karachi-75,270, Pakistan (voucher specimen: KU/BCH/SAQ/02).

### Reagents

Chloroform (Cat. no: 102447), glucose (Cat. no: D9434, dextrose), and trichloroacetic acid (TCA, Cat. no: T6399) were obtained from Merck & Co (New Jersey, US). Hexane (Cat. no: 296090, ethanol (Cat. no: V001229), DMSO (Cat. no: 276855), STZ (Cat. no: S0130), ketamine (Cat. no: K1884), xylazine (Cat. no: X1126), gliclazide (Cat. no: G2167), hydrochloric acid (HCl, Cat. no: 320331), hydrogen peroxide (H_2_O_2_, Cat. no: H1009), Ellman’s reagent (DTNB, 5,5′-dithiobis nitrobenzoic acid, Cat. no: D8130), glutathione (Cat. no: G4251), sodium azide (Cat. no: 13412), sodium arsenate (Cat. no: A6756), perchloric acid (Cat. no: V001526), hydroxylamine reagent (Cat. no: 159417), dichromate-acetic acid reagent (Cat. no: 223964), epinephrine (Cat. no: E4250), thiobarbituric acid (TBA, Cat. no: T5500) and TRIzol® Reagent (Cat.no: T9424) were procured from Sigma Aldrich Corp (St. Louis, MO, USA).

### Extraction and fractionation

**Fig. 1:**
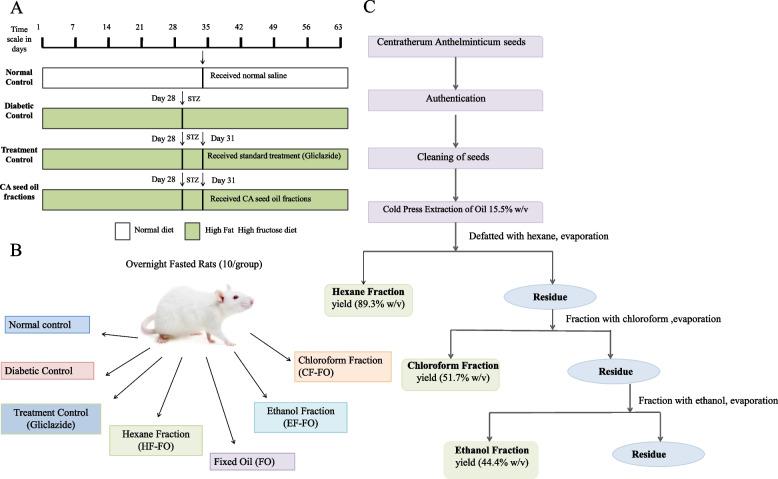
Schematic presentation of **A** experimental design of the study, **B** animal groups, and C cold press extraction of *C. anthelminticum* seed fixed oil (FO) followed by preparation of hexane (HF), chloroform (CF), and ethanol (CF) fractions of FO

The purchased and identified seeds were thoroughly cleaned and weighed. The fixed oil of seeds was extracted using the Cold-press method at low temperature (below 50 °C) [35]. The extracted fixed oil was defatted twice with hexane to obtain hexane soluble fraction and the hexane insoluble residues were fractionated with chloroform to obtain chloroform soluble and insoluble fractions, the insoluble fraction was further fractionated with ethanol. The obtained fractions (hexane, chloroform, and ethanol) were subject to evaporation, and concentration using Büchi Rotavapor R-200 (62-65 °C). The fractions were kept in separate small vials labeled as HF (Hexane fraction), CF (Chloroform faction), and EF (Ethanol fraction) for further use (Fig. 1C).

### Acute toxicity study

An acute toxicity study of *C. anthelminticum’s* FO and its fractions were performed on *Wistar* Albino rats of both sexes, aged 6-10 weeks after they were fasted for 14-16 hours. The study was conducted in compliance with the Organization of Economic Co-Operation and Development (OECD) guideline 420 for testing of chemicals [36]*.* The FO of *C. anthelminticum* and its fractions were dissolved in 0.05% dimethyl sulfoxide (DMSO) and orally administered once at a dose of 500, 1000, 1500, and 2000 mg/kg, to their respective to groups rats (*n* = 6; 3 males, 3 females); whereas the control group only received 0.05% DMSO (1 mL/kg) as a vehicle. The animals were allowed free access to water and food, they were followed for 24 hours with strict observation in the initial 6 hours and daily thereafter for 2 weeks for signs of acute toxicity. Once daily for 14 days, the animals were observed for changes in physical appearance, behavior, mortality, (i.e. salivation, lethargy, etc.), and acute illness/injury. On the 15th day, animals were euthanized through intraperitoneal injections of Ketamine 60 mg/kg and Xylazine 7 mg/kg body weight [37]. The cardiac puncture was performed on euthanized animals to collect blood in EDTA-containing (plasma) and non-heparinized (serum) vacutainer tubes for hematological (CBC, HbA1c) and biochemical (Urea, creatinine, and LFT) analysis, respectively.

### Oral glucose tolerance test (OGTT)

The experimental rats were divided into 7 groups each having 3 males and 3 females and they were fasted for 12 hours (for food). The animals were divided into control (glucose 2 g/kg), negative (glucose 2 g/kg + DMSO1mL/kg), and positive control (glucose 2 g/kg + standard drug: Gliclazide 200 mg/kg), and treatment groups divided on the basis of doses of FO and its fractions mentioned below).

Five doses (50, 100, 200,400, and 600 mg/kg) of each of *C.anthelminticum* FO and its fraction (HF, CF, and EF) were orally administered to their respective groups followed by a glucose load of 2 g/kg. Blood from the tail veins of rats was used to evaluate glucose levels at various time intervals (0, 30, 60, and 120 minutes) using a glucometer (ACCU-CHEK Roche, Switzerland) [38]. Upon completion of the OGTT study percent glycemic change between the control and test, groups were calculated [39].

### Animals

Male Albino *Wistar* rats (*n* = 65, body weight = 180-280 ± 20 g) were procured from DUHS (Dow University of Health Sciences, Karachi). Polycarbonate cages were used to house the rats individually; they were acclimated to 12 hours of light and dark cycle for a week at a 22 ± 3 °C temperature and 50 ± 10% humidity. During the acclimatization period and before dietary intervention rats had free access to sterilized water and a standard rat diet. The Ethical Review Board for Animal Research and Ethics, Dow University of Health Sciences approved the study (AR.IRB-21/DUHS/Approval/2021/037).

### Induction of diabetes in male Wistar rats

Male *Wistar* rats (10-12 weeks old; body weight 180-230 g) were separated into 6 treatment groups (*n* = 10 each) and a control group (*n* = 5). According to the groupings, the rats in the control group were fed with a normal diet whereas the ones in the other six treatment groups were fed a high-fat high-fructose (HF-HFr) diet for 28 days (i.e. 4 weeks). The HF-HFr diet used in this study mentioned in (Table 1) was the modification of protocol described by Yoo S et.al [40]. At the end of the 28th day, a single dose of STZ (60 mg/kg) in a citrate buffer (0.1 M, pH 4.5) was injected intraperitoneally into the 12-hour fasted rats in the treatment groups to develop T2DM. On the third day (i.e.72 hours) after STZ injection, FBG levels were measured from the tail vein of each rat using a glucometer, and rats having (FBG) levels of 230 mg/dL and above were considered as diabetic and randomly divided into 6 treatment groups (Fig. 1A).

**Table 1:** Composition and ingredients of experimental diet. RD: rats received a regular diet and 30 Frc + 45 Fat: rats received a 45 kcal% fat with a 30% fructose diet

%	RD		30 Frc		45 Fat		30 Frc + 45 Fat	
	g	kcal	g	kcal	g	kcal	g	kcal
Protein	20	20	20	20	24	20	24	20
Carbohydrate	64	64	64	64	41	35	41	35
Fat	7	16	7	16	24	45	24	45
Total		100		100		100		100
kcal/gm	4		4		4.8		4.8	
Ingredient	g	kcal	g	kcal	g	kcal	g	kcal
Casein, 80 Mesh	200	800	200	800	200	800	200	800
Soybean Oil	70	630	70	630	26	234	26	234
L-Cystine	3	12	3	12	3	12	0	0
Lard	0	0	0	0	174	1566	174	1566
Corn Starch	397.5	1590	229.5	918	137	548	0	0
Mineral Mix	35	0	35	0	35	0	0	0
Maltodextrin 10	132	528	100	400	100	400	37	148
t-Butylhydroquinone	0.014	0	0.014	0	0.014	0	0	0
Sucrose	100	400	0	0	100	400	0	0
Cellulose	50	0	50	0	50	0	50	0
Fructose	0	0	300	1200	0	0	300	1200
Choline Bitartrate	2.5	0	2.5	0	2.5	0	0	0
Vitamin Mix	10	40	10	40	10	40	10	40
Total	1000	4000	1000	4000	837.5	4000	797	3988

### Experimental design

Sixty-five male *Wistar* rats were divided into seven groups: 10 animals in each group, except the normal control group (5 animals). The *C. anthelminticum* FO and its fractions were administered orally at a dose of 200 mg/kg to treatment groups from day 31-63 (i.e. 4 weeks). The dosage for *C. anthelminticum* oil was calculated on the basis of the acute toxicity study and oral glucose tolerance test results. The treatment was given daily for 4 weeks. During the study, body weight and FBG were measured weekly using a weighing machine and ACCU-CHEK glucometer, respectively.

Group 1(NC) - Normal control rats; normal diet and treated with distilled water (1 mL/kg).

Group 2 (DM Control) - Diabetic control rats; rats were fed HF-HFr diet and were administered 60 mg/kg of STZ and orally administered 0.01% DMSO (1 mL/kg).

Group 3 (DM Glic); Diabetic rats (fed HF-HFr diet and administered 60 mg/kg of STZ) were treated with the reference drug; Gliclazide (200 mg/kg).

Group 4 (FO); rats with DM (fed HF-HFr diet and administered 60 mg/kg of STZ) were treated with FO (200 mg/kg) of *C. anthelminticum* seeds.

Group 5 (HF); rats with DM (fed HF-HFr diet and administered 60 mg/kg of STZ) were treated with HF (200 mg/kg) of *C. anthelminticum* seed oil.

Group 6 (CF); rats with DM (fed HF-HFr diet and administered 60 mg/kg of STZ) were treated with CF (200 mg/kg) of *C. anthelminticum* seed oil.

Group 7 (EF); rats with DM (fed HF-HFr diet and administered 60 mg/kg of STZ) were treated with EF (200 mg/kg) of *C. anthelminticum* seed oil.

### Biochemical analysis

On the 63rd day, the animals were sacrificed by intraperitoneal injection of Xylazine 7 mg/kg and Ketamine 60 mg/kg [37]. The blood samples were collected by cardiac puncture and centrifuged at 2000 x g for 15 minutes to separate serum for biochemical analysis. Serum insulin concentrations were determined according to the manufacturer’s instructions using a rat enzyme-linked immunoassay (ELISA) test kit (Bioassay Technology Laboratory Insulin ELISA kit Catalog no e0707RA). Glycated hemoglobin (HbA1c) and renal function assessment biomarkers such as serum creatinine and urea (mg/dL) were evaluated using commercially available spectrophotometric assay kits (Atellica Solutions, Siemens Healthcare). Homeostatic Model Assessment of Insulin Resistance (HOMA-IR), pancreatic β-cell function (HOMA β) and insulin sensitivity were calculated using the formulae: HOMA-IR = fasting insulin (μU/mL) × fasting glucose (mmol/L)/22.5 [41], HOMA β-cell (20 x insulin U/L/blood glucose - 3.5) and Insulin sensitivity = 1/log (fasting insulin U/L) x log (Fasting glucose mg/dl), respectively [41, 42]. The kidney tissues were excised, washed with ice-cold saline, and preserved in formalin 10% and phosphate buffer saline (PBS) for histopathological and PCR analysis, respectively.

### Histopathology of renal tissues

After animals were sacrificed, the collected renal tissue was harvested, sectioned longitudinally, and fixed with 10% neutral buffer formalin for 48 hours. Followed by dehydration with gradient alcohol and transparentize with xylene, waxed, embedded, and sectioned. The 3 to 4 μm thick sections were Hematoxylin-Eosin (H&E) stained for general morphological analysis. The pathological changes in the kidney were observed under a compound microscope [43].

### Homogenate preparation of renal tissue

All the tissues excised from both the control and experimental rats were placed in PBS and kept at − 80 °C. For homogenization, a 100 mM phosphate buffer with neutral pH was used. After complete tissue homogenization, the clear solution was centrifuged at 10,000×*g* for 15 minutes in order to remove any debris. The collected supernatant was used for further experimentation.

### Determination of renal lipid peroxidation (LPO)

The reagent TBA:HCl:TCA (15%:0.2 N:0.37%) was mixed with the kidney homogenate with a ratio of 1:1:1 (v/v). The mixture was then heated in boiling water for 15 minutes and was brought to room temperature for centrifugation at 5000 x g for 5 minutes. The absorbance was taken at 553 nm along with blank and the percent inhibition was calculated [44].

### Determination of renal superoxide dismutase (SOD) activity

The enzymatic activity of the superoxide dismutase (SOD) was determined by Misra and Fridovich, 1972 [45]. The prepared homogenate was mixed with 0.3 mM of freshly prepared epinephrine and 0.05 M carbonate buffer (pH 10.2). The absorbance was calculated at 480 nm every 30s for 150 s. The 50% inhibition of the rate of autoxidation of epinephrine measured as a change in absorbance /min was employed in calculating one unit of enzyme activity.

### Determination of renal catalase (CAT) activity

The catalase activity in the supernatant of kidney homogenate was assayed spectrophotometrically at 620 nm as described by Sinha [46]. The reaction mixture (1.5 mL) consisted of 0.1 mL of supernatant of kidney tissue homogenate, 0.4 mL of 2 M H_2_O_2,_ and 1.0 mL of 0.01 M pH 7.0 phosphate buffer. The 2 mL of dichromate-acetic acid reagent (5% potassium dichromate and glacial acetic acid were mixed in a 1:3 ratio) was added to the solution to stop the reaction and the absorbance was measured.

### Determination of renal HMG-CoA reductase activity

The activity of HMG-CoA reductase was determined in terms of the HMG-CoA/mevalonate ratio in kidney homogenate. The kidney homogenate was prepared in sodium arsenate solution. The homogenate was taken with an equal volume of dilute perchloric acid (PCA) mixed and incubated for 5 minutes at room temperature followed by a centrifuge at 3000 rpm for 10 minutes. 1.0 mL of kidney supernatant was collected in each of the two test tubes and allowed to react with 1.5 mL of ferric chloride and 0.5 mL of 2 M hydroxylamine reagent (alkaline pH = 5.5 in case of HMG-CoA and acidic pH = 2.1 in case of mevalonate) and incubated for 10 min. Absorbance was determined at 540 nm followed by a calculation of the HMG-CoA/mevalonate ratio [47].

### Determination of renal reduced glutathione (GSH) level and glutathione peroxidase (GPx) activity

The GSH levels in the kidney homogenate was determined by using the procedure of Ellman (1959). Kidney homogenate (1.0 mL) was mixed with 0.1 mL of 25% TCA and the precipitate was removed by centrifuge at 5000 x *g* for 10 min. 0.1 mL of supernatant was removed and added to 2 mL of 0.6 mM DTNB (5,5′-dithiobis nitrobenzoic acid) prepared in 0.2 M sodium phosphate buffer (pH 8.0). The absorbance was read at 412 nm [48].

GPx activity was measured by the method described by Rotruck et al*,* 1973. The reaction mixture contained 0.2 mL of 0.4 M Tris-HCl buffer pH 7.0, 0.1 mL of 10 mM sodium azide, 0.2 mL of tissue homogenate (homogenized in 0.4 M, Tris-HCl buffer, pH 7.0), 0.2 mL glutathione, and 0.1 mL of 0.2 mM hydrogen peroxide. The contents of the mixture were incubated at 37 °C for 10 min. The reaction was arrested by 0.4 mL of 10% TCA and centrifuged. The supernatant was assayed for glutathione content by using Ellman’s reagent (19.8 mg of 5,5′-dithiobis nitrobenzoic acid (DTNB) in 100 mL of 0.1% sodium nitrate) [49].

### Determination of levels of NF-κB p65 DNA binding activity

The ELISA of transcription factor NF-κB was carried out on the renal tissues homogenates as per manufacturer instruction (USCN Catalog no. SEB824Ra). The optical density of protein-bound NF-κB was measured at 450 nm.

### Reverse transcription quantitative real-time PCR (RT-qPCR) analysis

The kidneys of dissected animals were stored in PBS solution at − 80°C to preserve the RNA integrity. Total RNA was extracted using the TRIzol® Reagent. The integrity of the RNA was checked on 1% agarose gel electrophoresis. The quantitation of RNA was done with a nanodrop. Afterward, the complementary DNA (cDNA) was synthesized using a Thermo Scientific RevertAid First Strand cDNA Synthesis Kit (Catalog no. K1691 Thermofisher Scientific, USA). The PCR cDNA with eight different sets of primers was carried out using SYBR™ Green PCR Master Mix (Applied Biosystems, Foster City, CA, USA) and was performed in StepOnePlus Real-time PCR system. Table 2 contains the primer sets used in RT-qPCR. The gene HPRT-1 was used as a reference gene to measure the relative expression of the mRNA in a sample. The amplification PCR program included 1 cycle of 94 °C for 10 min, followed by 35 cycles of 94 °C for 1 min, 60 °C for 40 sec, and 72 °C for 30 sec, and a final elongation cycle at 72 °C for 5 min using an Applied Biosystems, Foster City, CA, USA. Each sample was run in duplicates in order to ensure the reproducibility of the reaction [32].

**Table 2:** Forward and reverse primers (5′ → 3′) for reverse transcriptase-real time PCR (RT-qPCR)

Sr.no	Gene name	Forward primer	Reverse primer
1	IL-1β	CAGCTTTCGACAGTGAGGAGA	GTCGAGATGCTGCTGTGAGA
2	Nrf-2	GCAAGAGACTTCCAGCCAGT	TGCCATTGCACAACTCTTTTC
3	TNF-α	TTCTCATTCCTGCTCGTGGC	CTCCGCTTGGTGGTTTGCTAC
4	HO-1	CAGAACCCAGTCTATGCCCC	TGTGTGGCTGGTGTTAAGGG
5	Keap1	CTGTGACACTTCTCCTGGGG	GAGAAGCAGGAACCAGGCAT
6	HPRT1	AGTCCCAGCGTCGTGATTAGT	CCAGCAGGTCAGCAAAGAAC
7	Bax	CACGTCTGCGGGGAGTCA	CTCGATCCTGGATGAAACCCT
8	Bcl2	CTTTGAGTTCGGTGGGGTCA	AGTTCCACAAAGGCATCCCAG
9	COX-1	AACCGTGTGTGTGACTTGCT	TTGCGATACTGGAACTGGGC

### Statistical analysis

The results are expressed as the mean ± standard error mean (S.E.M). Statistical analysis was carried out using the One-way ANOVA followed by least significant difference (LSD) multiple comparisons post-hoc test. A value of *p* < 0.05 was considered statistically significant. IBM SPSS v. 26 software (Chicago, IL, USA) was used for statistical analysis.

## Results

### Acute toxicity of C. anthelminticum fixed oil and its fractions

No toxicity and mortality due to *C. anthelminticum* fixed oil and its hexane, chloroform, and ethanol fractions were observed within 24-48 hours and for 15 days thereafter. All the administered doses were found to be safe up to 2000 mg/kg (Supplementary File 1).

### Effect on Oral glucose tolerance test

**Fig. 2:**
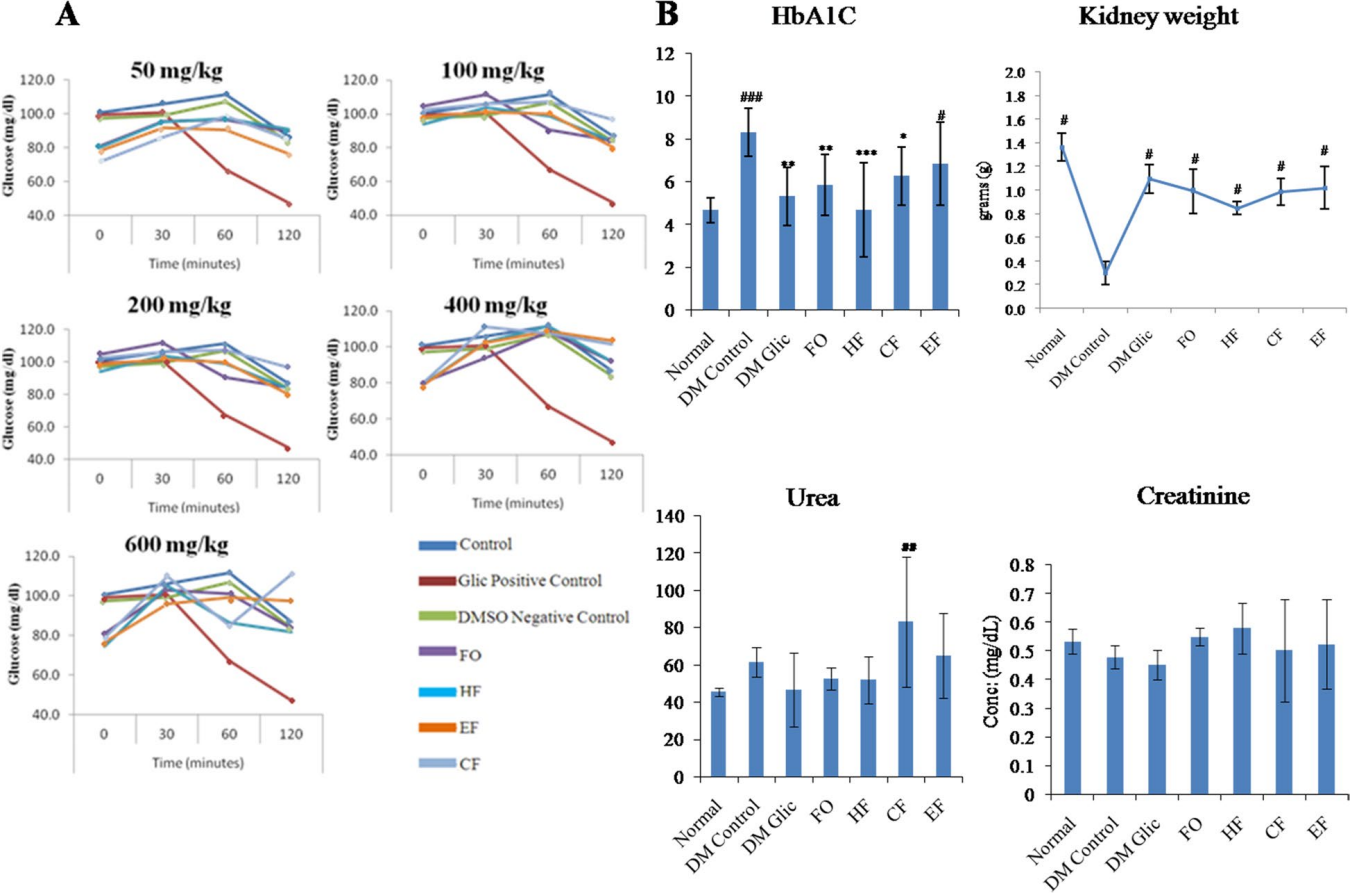
**A** Oral glucose tolerance test of *C. anthelminticum* seed FO and its fractions was carried out at doses of 50, 100, 200, 400 and 600 mg/kg followed by glucose load 2 g/kg and was compared with control (glucose 2 g/kg), Glic positive control (gliclazide 200 mg/kg + glucose 2 g/kg) and DMSO negative control (0.05% DMSO 1 mL/kg + glucose 2 g/kg). **B** The effects of *C. anthelminticum* FO and its fractions on HbA1c, kidney weight, serum urea, and creatinine in HF-HFr-STZ induced diabetic rats. The data are expressed as the mean ± S.E.M. Statistical analysis was carried out using the One-way ANOVA followed by LSD multiple comparisons post-hoc test. *p < 0.05 versus Normal; #*p* < 0.05 versus DM Control

In OGTT, 200 mg/kg of *C. anthelminticum* fixed oil and its fractions produced hypoglycemic state in rats challenged with a glucose load of 2 g/kg. The maximum reduction in blood glucose was observed between 30 to 60 minutes whereas between 60 to 120 minutes glucose levels were maintained within euglycemic levels as compared to the standard anti-diabetic drug (Gliclazide). A reduction in the concentration of blood glucose was found in all doses of *C. anthelminticum* fixed oil and its fractions from 50 to 600 mg/kg (*p* < 0.05) after 120 min (Supplementary File 2). Based on the present acute toxicity study (Fig. 2A), OGTT and previous studies of *C.anthelminticum* seed extract in rabbits, rats, and humans, 200 mg/kg was selected as a final dose of intervention [39, 50–53].

### Effect on fasting blood glucose, serum insulin, HOMA-IR, HOMA-β, and insulin sensitivity (IS)

Table 3 shows the level of FBG and serum insulin in rats from normal, diabetic, and treatment groups. Both insulin resistance and elevated blood glucose level are the initial indicators of HF-HFr diet-induced T2DM followed by administration of a single dose of STZ (60 mg/kg). The level of blood glucose significantly increased in the diabetic control group compared to the normal control (*p* < 0.05); whereas the serum insulin in diabetic rats as compared to control rats decreased (*p* < 0.05). Significant improvement in insulin levels and reduction in blood glucose was recorded in all the four diabetic treatment groups (FO, HF, CF, and EF) when compared to the diabetic control groups. Furthermore, the results shown in Table 3 signify that the HOMA-IR index was significantly (*p* < 0.05) higher in the DM-C group when compared to the normal, treatment control and *C. anthelminticum* treated groups. Similarly, significant (*p* < 0.05) differences were observed between diabetic control and the *C. anthelminticum* seed fixed oil group. In the diabetic control group, the β cell functioning index and insulin sensitivity (IS) were significantly compromised when compared to the normal group and the treated groups.

**Table 3:** Quantification of FBG, serum insulin, insulin sensitivity, resistance, and beta-cell function in T2DM rats treated with FO and its HF, CF, and EF (200 mg/kg) for 28 days

Groups	FBS (mg/dL)	Serum Insulin (mIU/L)	HOMA-IR	HOMA-B	Insulin sensitivity
Control	83.1 ± 13*	9.24 ± 0.17*	1.59 ± 0.12	44.1 ± 3.45	1.76 ± 1.0
DM Control	270.6 ± 33	7.46 ± 0.06	4.8 ± 0.20	6.82 ± 0.42	0.98 ± 0.02
DM Glic	146.7 ± 98*	11.6 ± 0.48	2.43 ± 0.78*	48.5 ± 18.35*	1.44 ± 0.28*
HF	97.25 ± 29*	11.85 ± 1.61	3.8 ± 2.77	40.2 ± 25.31*	1.27 ± 0.39
EF	105.2 ± 37*	11.12 ± 0.96	5.36 ± 2.09	19.7 ± 11.5	0.96 ± 0.15
CF	182.8 ± 62*	11.15 ± 1.62	3.03 ± 1.06	37.1 ± 17.8*	1.27 ± 0.23
FO	82.3 ± 17*	10.8 ± 0.37*	1.82 ± 0.07*	53.4 ± 1.22*	1.67 ± 0.05*

* shows the significant difference of *p-value* < 0.05 as compared to DM control

### Effect on hemoglobin A1c level

The level of HbA1c level had increased in STZ-induced diabetic control rats whereas *C. anthelminticum* seed FO, CF, and HF treated groups (200 mg/kg b.w) exhibited a significant (*p* < 0.05) decline in glycated hemoglobin as compared to EF group in which there is no significant decrease found (Fig. 2B).

### Effect on biochemical parameters and kidney weight

In this study, the renal function parameters including serum creatinine and urea were measured using commercial assay kits. Data shows higher levels of urea in diabetic control rats and rats treated with chloroform fraction of seed oil than those in normal and other treatment groups (i.e. FO, HF, and EF) whereas; no significant change was observed in creatinine levels in either diabetic or treatment groups. However, the diabetic control group exhibited a decrease in kidney weight as compared to the normal. On the other hand treatment with *C. anthelminticum* FO and its fraction reinstated the kidney mass to near normal (Fig. 2B).

### Effect of C. anthelminticum fixed oil and its fraction on renal histology

**Fig. 3:**
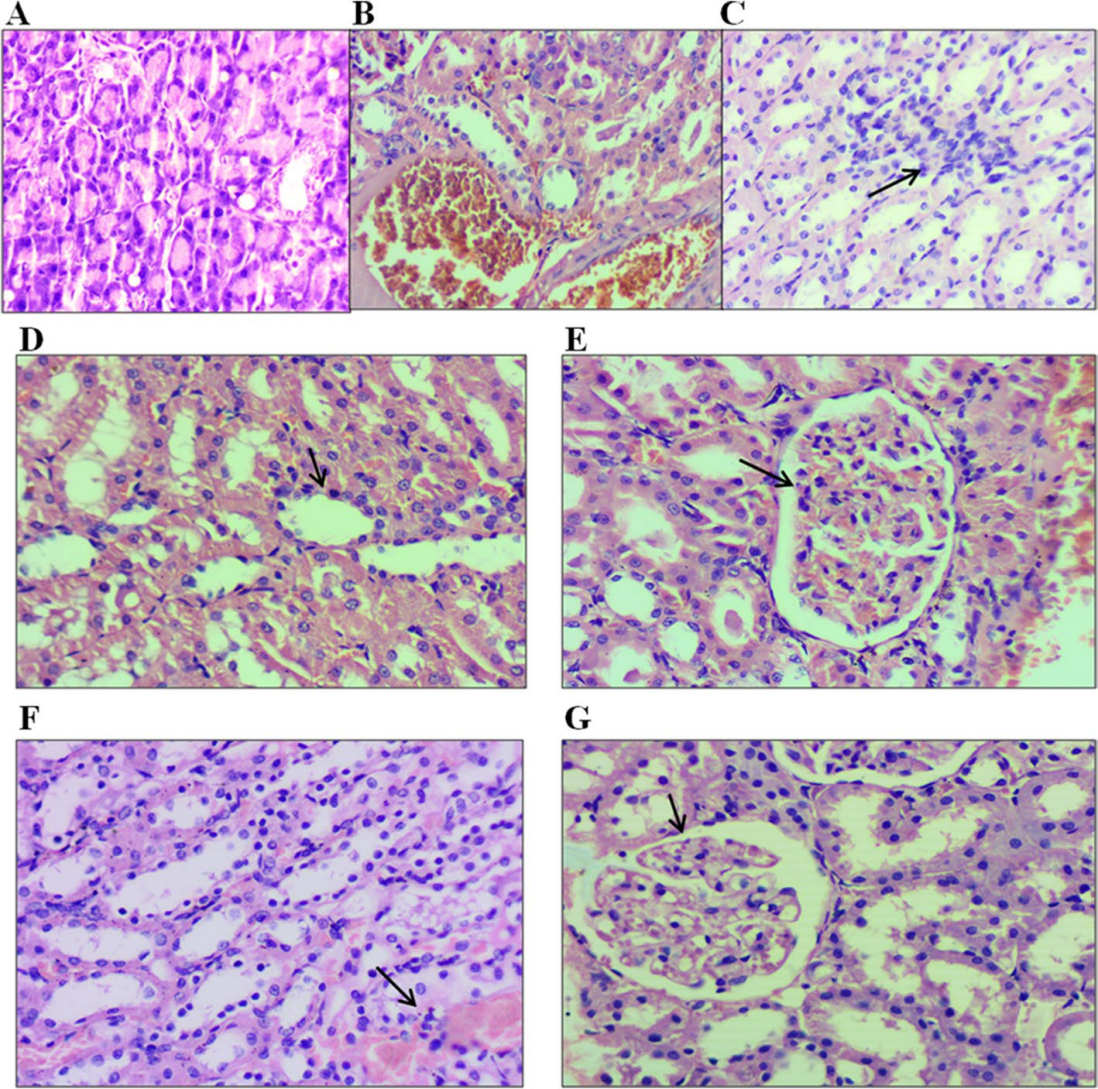
Histopathological changes in HF-HFr-STZ induced diabetic rat kidneys treated with *C. anthelminticum* fixed oil and its fractions. The figure shows a photomicrograph of (A) Normal control (NC) kidney showing normal architecture, (B) Diabetic control (DM-C) showing congestion of blood with signs of inflammation, and (C) Treatment control (DM Glic) reveals attenuation in inflammation as compared to DM-C; and (D, E, F, and G) are the photomicrograph of *C. anthelminticum* fixed oil and HF, CF, and EF-treated kidney, respectively. Degeneration and inflammation observed in DM-C have subsided upon treatment with *C. anthelminticum* seed oil and its fractions. Renal cell architecture is near normal

Morphological changes in renal tissue of normal control rats determined using H&E staining revealed normal renal cortex and parenchyma with normal glomeruli and renal tubules (Fig. 3). The photomicrographs of kidneys from the HF-HFr-STZ-induced diabetic rats demonstrated interstitial hemorrhage, degenerative changes, and focal inflammatory cell infiltration, whereas 4 weeks of treatment with *C. anthelminticum* FO and its fractions at a dose of 200 mg/kg improved the architecture of renal tissue.

### C. anthelminticum fixed oil and its fractions mitigate diabetes-induced renal oxidative stress

**Fig. 4:**
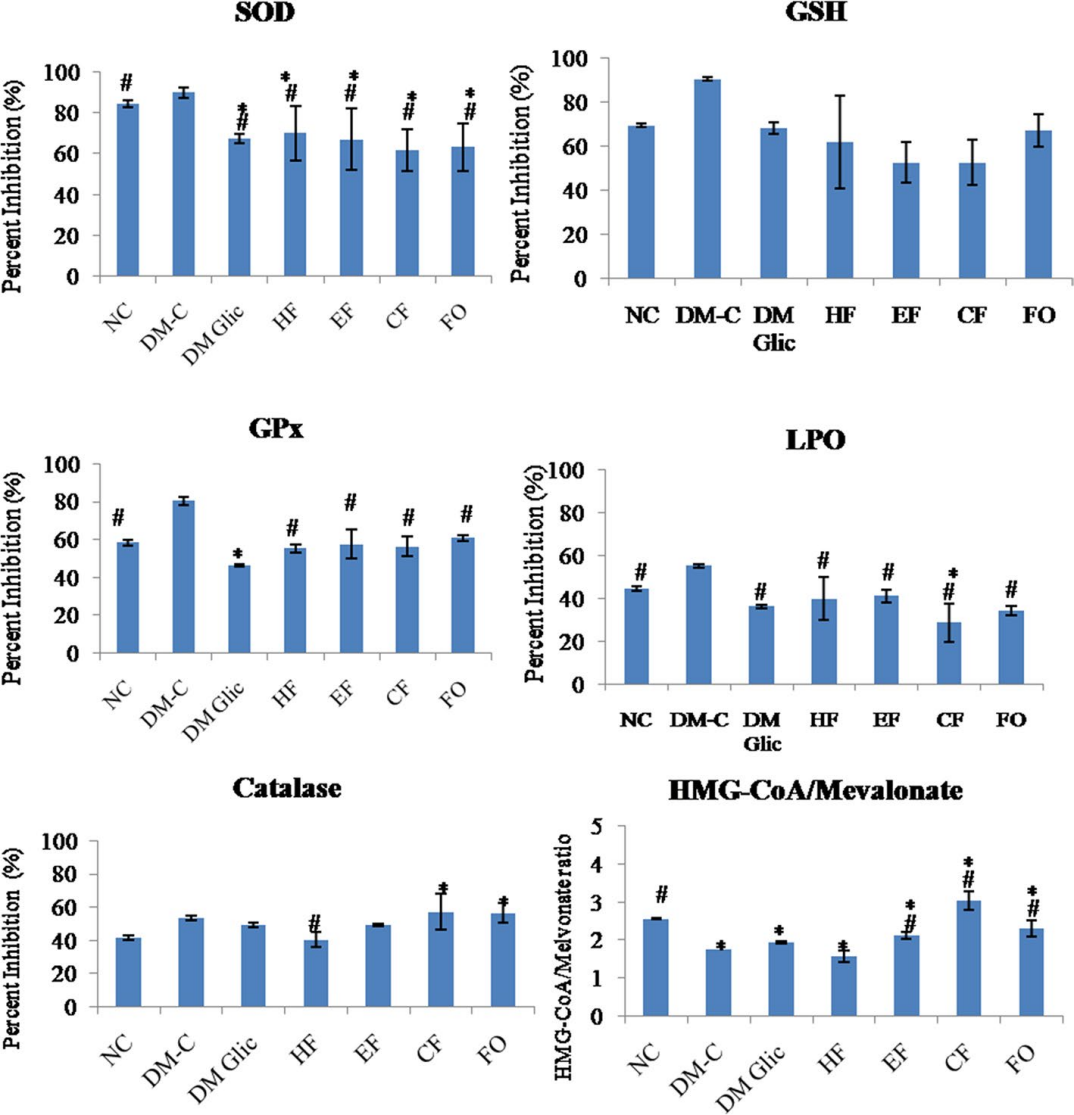
Administration of *C. anthelminticum* FO and its fractions alleviate oxidative stress in DN. The percent inhibition of SOD, reduced GSH, GPx, CAT, and HMG-COA: Melvonate ratio except LPO in kidney tissue extracts of different groups. The results are expressed as the mean ± S.E.M. Statistical analysis was carried out using the One-way ANOVA followed by LSD multiple comparisons post-hoc test. Abbreviations: DM-C: DM + 1 mL/kg distilled water, DM-Glic: DM + 200 mg/kg Glicalized, FO: DM + 200 mg/kg *C. anthelminticum* fixed oil, HF: DM + 200 mg/kg hexane fraction, CF: DM-control + 200 mg/kg chloroform fraction and EF: DM + 200 mg/kg ethanol fraction. *p < 0.05 versus Normal; #*p* < 0.05 versus DM Control

The biological activities of SOD, CAT, GPx, and GSH levels in the kidney of HF-HFr-STZ diabetic rats decreased significantly (*p* < 0.05) by showing increased percent inhibition of.

these enzymes when compared to control rats (Fig. 4). Oral administration of 200 mg/kg of *C. anthelminticum* fixed oil and its fractions (i.e. HF, CF, and EF) raised the activities of these enzymes (SOD, CAT, GPx) and GSH significantly (p < 0.05) in their respective groups by displaying their less percent inhibition when compared with the diabetic control group (Fig. 4).

### Effect of C. anthelminticum fixed oil and its fractions on LPO and HMG-CoA reductase/mevalonate ratio

The level of malondialdehyde (MDA) which is the end product of LPO was significantly decreased in kidney tissue homogenate of the diabetic control group as compared to the normal control (Fig. 4). After 4 weeks of *C. anthelminticum* fixed oil and its fractions treatment; the MDA level in the treatment groups increased, and a significant difference was observed between the DM control group vs. treatment groups (*p* < 0.05). The HMG-CoA/Mevalonate ratio improved in FO, EF, and CF groups as compared to DM-control (*p* < 0.05) (Fig. 4).

### C. anthelminticum fixed oil and its fractions ameliorate diabetes-induced renal inflammation

**Fig. 5:**
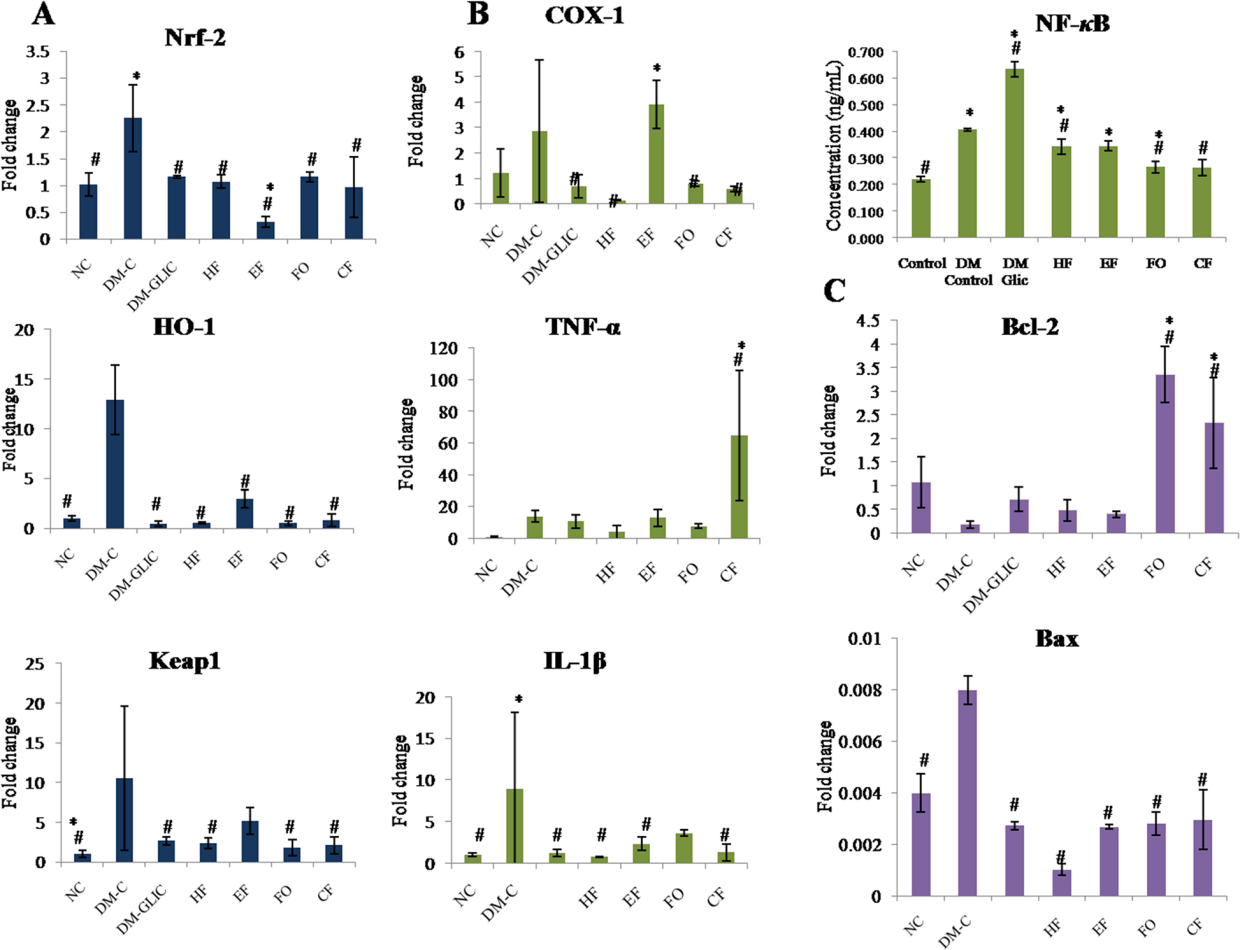
RT-qPCR showing cytosolic expression of (A) Nrf-2/Keap1/HO-1-mediated antioxidant pathway, (B) inflammatory (IL-β and TNF-α) and NF-κB (ELISA) pathway, and (C) apoptotic (Bcl-2 and Bax) pathway in the kidney homogenate from diabetic kidney treated with *C. anthelminticum* fixed oil and its fractions. The values are expressed as the mean ± S.E.M. Statistical analysis was carried out using the One-way ANOVA followed by LSD multiple comparisons post hoc test, *p < 0.05 versus Normal Control (NC); #*p* < 0.05 versus DM Control. Abbreviations: DM-C: DM + 1 mL/kg distilled water, DM-Glic: DM + 200 mg/kg Glicalized, FO: DM + 200 mg/kg *C. anthelminticum* fixed oil, HF: DM + 200 mg/kg hexane fraction, CF: DM + 200 mg/kg chloroform fraction and EF: DM + 200 mg/kg ethanol fraction

In order to analyze the anti-inflammatory potential of *C. anthelminticum* fixed oil and its fractions, a pro-inflammatory NF-κB mediated pathway was explored. The relative mRNA expression of inflammatory markers IL-1β, TNF-α, and COX-1 were assessed by reverse transcriptase real-time PCR whereas; NF-κB p65 protein was measured using ELISA. The expression and protein levels of pro-inflammatory markers: IL-1β, TNF-α COX-1, and NF-κB p65, respectively were significantly increased in diabetic rats. Diabetic rats treated with Gliclazide, *C. anthelminticum* seed FO, and its fractions (i.e. HF, CF, and EF) showed significant reduction (*p* < 0.05) in the expression of IL-1β whereas; the levels of TNF-α decreased but not significantly. Similarly, COX-1 showed a reduction (*p* < 0.05) compared to diabetic control. The level of pro-inflammatory transcription factor NF-κB p65 improved with treatment (*p* < 0.05) as shown in Fig. 5.

### C. anthelminticum fixed oil and its fractions prevent renal apoptosis

The mRNA expression level of pro-apoptotic Bax and anti-apoptotic Bcl-2 markers are illustrated in Fig. 5. The expression levels of Bax were elevated, whereas; Bcl-2 mRNA levels were found to be reduced in diabetic control rats when compared to normal rats. Following treatment, the levels of Bax were significantly down-regulated in treatment control (Gliclazide) as compared to all the treatment groups (*p* < 0.05). Compared to diabetic control, Bcl-2 expression levels were up-regulated in treatment groups (Fig. 5C).

### Antioxidant role of C. anthelminticum fixed oil and its fractions in protecting renal tissue through Nrf-2/Keap1/HO-1 pathway

Oxidative stress along with inflammation lies at the crux of DN pathogenesis. Nrf-2/keap1/HO-1 being a major defense mechanism against oxidative insult was evaluated to understand its role in the progression and development of DN. Expression levels of Nrf-2, keap1, and HO-1 were measured in all the study groups. The levels of all the three antioxidant markers were significantly elevated (*p* < 0.05) in the diabetic control group in comparison to the normal control (Fig. 5). *C. anthelminticum* FO and its fraction (i.e HF, CF, and EF) administered to treatment groups showed a decrease in the expression of Nrf2, Keap1, and HO-1 as compared to the diabetic group (*p* < 0.05). The most significant decrease was observed in EF and CF (*p* < 0.05). The expression level of antioxidant markers in the treatment groups were near to normal as observed in the normal control group (Fig. 5A).

## Discussion

Prolonged redox imbalances manifesting both as chronic oxidative and inflammatory stress in DM delineate the role of the same in the pathophysiology and progression of its most debilitating complication; diabetic nephropathy [54, 55]. Most cited literature highlights its pathophysiology in the context of chronicity but how the kidney tries to compensate in the early stage and what the consequences of early intervention with strategies other than the existing conventional one are inadequate. *C. anthelminticum* seed’s known antihyperglycemic, anti-inflammatory, and antioxidant activities are likely to have the potential to help in the prevention and progression of T2DM [56, 57].

Innately at cellular and molecular levels, antioxidant/anti-stress systems exist to try to mitigate the oxidative and inflammatory responses. The key player of this system is nuclear factor erythroid 2-related factor 2 (Nrf-2); a transcription factor that forms a complex with its substrate adaptor Kelch-like ECH-associated protein 1 (Keap1) with Cullin (Cul3)-containing E3 ubiquitin ligase and is found in the cytoplasm [58–60]. Under basal conditions, Nrf-2 is sequestered by Keap1 and degraded by E3 ubiquitin ligase [61]. In the face of xenobiotic, oxidative, electrophile, and metabolic stress Nrf-2 escapes from the Keap/Cul3-Rbx1 ubiquitination in a dose-dependent manner and the free (or newly) synthesized Nrf-2 translocates into the nucleus [61, 62]. Inside the nucleus, Nrf-2 heterodimerizes with one of the small Maf (musculoaponeurotic fibrosarcoma oncogene homolog) protein to recognize the enhancer sequence called antioxidant response element (ARE) present in its regulatory region [63]. It has been cited in the literature that Nrf-2 is involved in the expression of nearly 500 genes involved in redox homeostasis, detoxification, anti-stress/anti-oxidation, and indirectly/directly anti-inflammation activities [64, 65].

In the context of scientific evidence and findings from the experimental animal models of DN, it is observed that Nrf-2 adapts to changing oxidative and inflammatory stress by trying to not only remain functional rather, also increase its expression levels. This adaptation is to overcome the glucolipotoxicity insult faced in the initial stages [66]. Jiang et al. has cited the similar findings [58]; they have also proposed that up-regulation and activation of Nrf-2 during the early stages of kidney insult is an attempt of innately existing antioxidant/anti-stress mechanisms to prevent the progression of DN. However, in the face of persistent glycemic, oxidative, and inflammatory insults, the protective mechanisms become saturated with excessive ROS, and the kidney insult progresses to advanced stages of ESRD [58]. It means the high and low expression levels of Nrf-2 parallel the early and advanced stages of DN. Furthermore, a redox-regulated transcription factor NF-κB, after its translocation inside the nucleus increases the expression of pro-inflammatory cytokines: TNF-α, IL-6, IL-1β, COX-1, and COX-2. Rather, in the light of the emerging evidences it has been shown to facilitate Nrf-2 activity with the help of a small Ras-related C3 botulinum toxin substrate 1 (Rac1) protein with GTPase activity [67]. Rac1 not only mediates activation and nuclear translocation of Nrf-2 in keap1 independent manner rather, also up-regulates HO-1 expression (regulated by Nrf-2) and suppresses activation of the NF-κB pathway. Up-regulation of HO-1 mediated by Nrf-2 and indirectly by Rac1, in turn, suppresses inflammatory activity mediated via NF-κB [68]. These cited findings suggest that Nrf-2 is a putative antioxidant target that could either prevent or slow the progression of DN. Therefore, a number of novel therapeutic molecules are undergoing clinical trials. Recently, bardoxolone methyl showed promising Nrf-2 stimulating activity however, the trial could not continue due to adverse cardiovascular events [69].

**Fig. 6:**
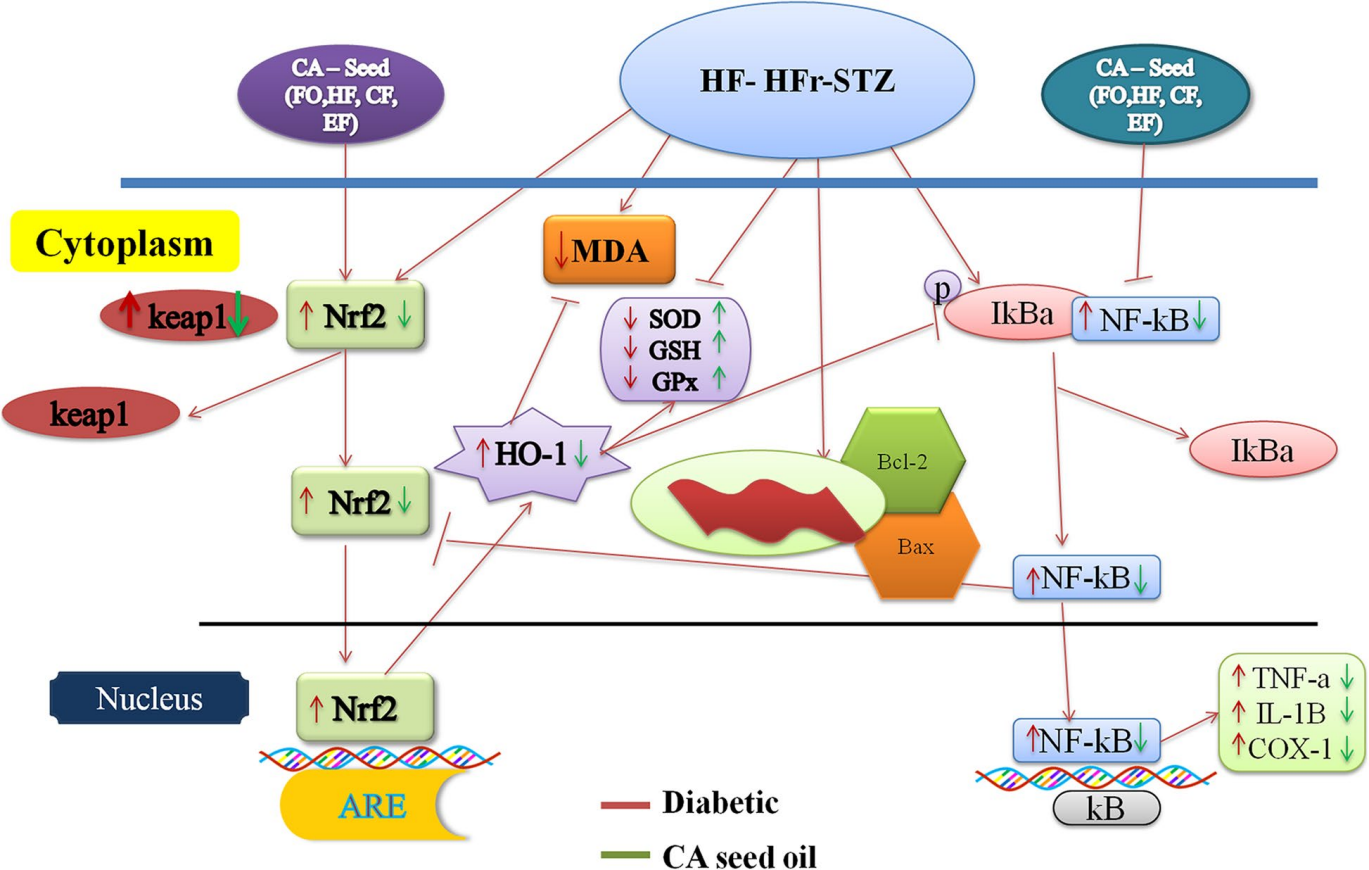
The schematic diagram is summarizing the actions of the intervention of *C. anthelminticum* seed oil on Nrf-2/HO-1 and NF-ĸB pathways. Upon exposure to oxidative, inflammatory, and glycemic stress expression levels of Nrf-2 increase and it translocates into the nucleus where it binds ARE to activate the expression of the HO-1 (antioxidant) gene (red arrow). Similarly, NF-ĸB is also translocated from the cytosol to the nucleus, where it activates the transcription of genes that include TNF-α, and IL-1β (red arrow). Following treatment with *C. anthelminticum* seed oil and its fractions, NF-ĸB activation is downregulated and it is reflected in the decrease in expression levels of Nrf-2/HO-1 to near normal (green arrow). Nrf-2-driven transcription of HO-1 is normalized which attempts to mitigate oxidative stress (green arrow). The perturbations of both NF-ĸB and Nrf-2/HO-1 signaling points to putative cross-talk occurring between the two. However, the mechanism of this process in kidney disease remains unknown

*C. anthelminticum* seeds fixed oil antioxidant activity in the context of Nrf-2 has not been explored. We, therefore, proposed to evaluate the anti-inflammatory, antihyperglycemic, anti-apoptotic, and antioxidant role of seed’s fixed oil and its fractions in the diabetic kidneys via modulation of Nrf-2. HF-HFr-STZ-induced hyperglycemia leads to the suppression of antioxidant activities of SOD, CAT, GPx, and GSH by showing high percent inhibition of these enzymes. The HMG-CoA:Mevalonate ratio was also found decreased in the same group (DM-C) as HF-HFr-STZ-induced hyperglycemia accelerates cholesterol biosynthesis as compared to other treated groups (EF, CF & FO). However, interestingly high percent inhibition of LPO was found in the DM-C group. In our study LPO for some reason did not occur which would normally be accepted to occur in diabetes-induced oxidative stress. The exhaustive activity of antioxidant enzymes (proteins) in tissue reflects the oxidative toll taken by the tissue which in turn is reflected upon the mRNA levels of transcriptional factor Nrf-2 and its downstream master regulator of antioxidant mechanisms: HO-1(Fig. 5A). Therefore, it can be speculated that if the expression levels of SOD and GPx are measured they might also be increased as they are controlled by Nrf2 and are involved in ROS catabolism [70, 71]. Similarly, the increasing level of NF-κB reflects inflammatory stress which is seen as increased levels of IL-1β, TNF-α, and COX-1 whereas; decreasing Bcl-2 and increasing Bax show apoptotic activity (Fig. 5B and C, *p* < 0.05). *C. anthelminticum* fixed oil reversed these deleterious effects and improved blood glucose which could be attributed to improved beta-cell function and insulin sensitivity without a decrease in serum insulin levels [39, 51, 52, 57, 72] (Table 3). The *C. anthelminticum* fixed oil and its fractions treatment also dampen the inflammatory damage and preserve the kidney structural damage observed at the tissue level (Figs. 5B and 3). These improvements could also be attributed due to its antioxidant potential. However, hyperglycemia, oxidative and inflammatory stress in our model increased the mRNA levels of Nrf-2, Keap1, and HO-1 (Fig. 5A); the increased Nrf-2 might be due to increased de novo synthesis (Fig. 6).

Dissecting the complex transcriptional activation and cellular expression of the Nrf-2 gene has delineated that its promoter region, *NFE2L2* in mice also contains the ARE sequence to which Nrf-2 may bind thus providing a positive feedback loop to not only amplify its effects rather its expression as well [73, 74]. Nrf-2 also regulates the transcription and expression of heme-oxygenase 1 (HO-1) therefore, is regarded as the master regulator of the oxidative stress response [75]. Furthermore, it has been cited by Rushworth, S. A *et.al;* that increasing levels of NF-κB, c-Jun, and c-Fos in response to inflammation activates the transcription of the NFE2L2 gene, mediating the increase in Nrf-2 expression [68]. Thus highlighting the putative cross-talk occurring between Nrf-2 and NF-κB signaling pathways in order to maintain not only cellular redox homeostasis but also regulate cells’ response to inflammatory insult and stress [76]. However, this functional cross-talk between the said signaling pathways appears to be tissue and cell-type-specific, and much needs to be explained and explored about their cellular and molecular interaction [77]. Very interestingly, the administration of *C. anthelminticum* seed oil and its fraction dampens the inflammation by decreasing the NF-κB which is also reflected in the expression of IL-1β, TNF-α, and COX-1 [67] (Fig. 5B). A decrease in inflammation simultaneously brought the expression profiles of Nrf-2, keap1, and HO-1 to near-normal constitutive levels as observed in the normal control model (Fig. 5A). This finding supports the notion of the molecular and cellular communication occurring between the Nrf-2- NF-κB pathways; both are either working for or against each other to restore the redox balance [68, 78, 79]. The model lasted for approximately 9 weeks; therefore, the findings of the study are representing more or less acute changes both biochemically and at the tissue level.

The study findings have highlighted the response of antioxidant pathways in the context of acute insults much in the same way when the body’s immune system reacts to acute infectious insults; the innate and reflexive defense mechanisms all go in a forward drive to correct the insult. This means an earlier intervention can be helpful to slow and even reverse the progression of DN. The observed increase in the expression levels of Nrf-2 in the diabetic model and near normalcy of Nrf-2, Keap1, and HO-1 by *C. anthelminticum* seed oil calls for exploring its nuclear translocation and nuclear to cytoplasmic ratio. Furthermore, its biphasic levels with upregulation in early phases and down-regulation when DN progresses to ESRD needs to be explored. Similarly, the parallel behavior of Nrf-2 and Keap1 in diabetic and treated rats needs to be delineated for a better understanding of the mechanisms.

For further understanding of how the Nrf-2 mRNA expression profile changes acutely with changes in glucose concentration in time; cells can be treated with increasing doses of glucose at different time intervals and this could be validated using experimental in-vivo models and measuring protein expression as well. Furthermore, for exploring mechanisms of *C. anthelminticum* seed oil both in the early and later stages of DN, transfection studies using validated Nrf-2 specific siRNA can be used for ex-vivo models along with in vivo models. This will help to understand whether *C. anthelminticum* oil is: (i) Nrf-2 nuclear translocation activator; (ii) inducing the de novo synthesis of Nrf-2; or (iii) it is disrupting the Nrf-2-Keap1 complex alone without the induction of Nrf-2 synthesis. Nrf-2-NF-κB communication needs to be dissected as well to further understand the mechanism of *C. anthelminticum* oil; as this could be the possibility that rather than activating Nrf-2 directly, it is targeting NF-κB signaling, which is conversely activating the Nrf-2 pathway in an acute state.

## Conclusion

This study explored the potential antioxidant, anti-inflammatory, and antihyperglycemic effects of the *C. anthelminticum* seeds fixed oil and its fractions. Despite the short intervention period of 4 weeks, the effects were promising and it can be said that seed oil has the potential to dampen inflammatory and oxidative stress in early DN. The fixed oil and its hexane fraction were found effective and had the satisfactory ability to reverse diabetic perturbations to near normal. The evidence cited in the literature and the results from this study give credibility to the valuable ethno-therapeutic properties of *C. anthelminticum*. The study also speculates the antinephropathic effect at an early stage can be attributed to its ability to down-regulate NF-κB which reflects by bringing the Nrf2 expression levels to near normal. Moreover, it can also have the potential of having significant long-term or chronic effects as well, which needs to be explored further. Our laboratory is presently working on the isolation and identification of the bioactive components from *C. anthelminticum* seed oil and its fractions for further evaluation.

## Supplementary Information

**Additional file 1.**

**Additional file 2.**

## Data Availability

The datasets used and/or analyzed during the current study are available from the corresponding author on reasonable request.

## Abbreviations

DM: Diabetes mellitus
IDF: International Diabetes Federation
T2DM: Type 2 Diabetes Mellitus
GFR: Glomerular filtration rate
PF: Polyherbal formulations
OECD: Organization of Economic Co-Operation and Development
DUHS: Dow University of Health Sciences
FBG: Fasting blood glucose
HOMA-IR: Homeostatic Model Assessment of Insulin Resistance
HOMA β: Pancreatic β-cell function
LPO: Lipid peroxidation
HF-HFr-STZ: High fat-high fructose-streptozotocin
TBA: Thiobarbituric acid
TCA: Trichloroacetic acid
HCL: Hydrochloric acid
HF: Hexane Fraction
EF: Ethanol Fraction
CF: Chloroform Fraction
FO: Fixed Oil
DPPH: 1,1-Diphenyl-2-Picrylhydrazyl
FRAP: Ferric reducing antioxidant power assay
