# Supplementary material for: Antioxidant and anti-inflammatory activities of Centratherum anthelminticum (L.) Kuntze seed oil in diabetic nephropathy via modulation of Nrf-2/HO-1 and NF-κB pathway
Source: BMC Complement Med Ther. 2022 Nov 18;22:301. doi: 10.1186/s12906-022-03776-x (PMC9675141; doi:10.1186/s12906-022-03776-x)
Supplement: Supplementary file 1 — Additional file 1. [file 12906_2022_3776_MOESM1_ESM.docx]

**ACUTE TOXICITY TEST**

| **No.** | **Response** | **Animals** | | | | | |
| --- | --- | --- | --- | --- | --- | --- | --- |
|  |  | **Before experiment** | **After experiment**  **500mg/kg** | **After experiment**  **1000mg/kg** | **After experiment**  **1500mg/kg** | **After experiment**  **2000mg/kg** | |
| 1. | Alertness | √ | √ | √ | √ | √ | |
| 2. | Restlessness | X | X | X | X | X | |
| 3. | Touch response | √ | √ | √ | √ | √ | |
| 4. | Torch response | √ | √ | √ | √ | √ | |
| 5. | Pain response | √ | √ | √ | √ | √ | |
| 6. | Sedative behavior | X | X | X | X | X | |
| 7. | Rash | X | X | X | X | X | |
| 8. | tremors | X | X | X | X | X | |
| 9. | Pigmentation | X | X | X | X | X | |
| 10. | Hair loss | X | X | X | X | X | |
| 11. | Food intake | √ | √ | √ | √ | √ | |
| 12. | Water intake | √ | √ | √ | √ | √ | |
| 13. | Mortality | Not applicable | X | X | Present | | Present |

The table shows the results of acute toxicity study of all C. anthelminticum seed oil and its fractions on varying doses.

**HEMATOLOGICAL PARAMETERS**

**FIXED OIL**

| Parameters | Control | Male  1000mg/kg | Female  1000mg/kg | Male  2000mg/kg |
| --- | --- | --- | --- | --- |
| HB | 12.5 gm/dL | 10.5 gm/dL | 9.3 gm/dL | 15.5 gm/dL |
| RBC | 5.9 x 10^12/L | 5.9 x 10^12/L | 4.9 x 10 ^^^12 | 8.1 x 10 ^^^12 |
| HCT | 34% | 36% | 31 % | 42 % |
| MCV | 57 fL | 61 fL | 63 fL | 52 fL |
| MCH | 21 pg | 18 pg | 19 pg | 19 pg |
| MCHC | 37 gm/dL | 29 gm/dL | 30 gm/dL | 37 gm/dL |
| WBC | 3.6 x 10e9/L | 3.1 x 10e9/L | 3.1 x 10e9/L | 3.9 x 10e9/L |
| Platelets | 931 x 10^9/L | 801 x 10^9/L | 474 x 10^9/L | 1073 x 10^9/L |
| Neutrophils % | 32% | 24 % | 21 % | 04 % |
| Lymphocytes % | 58% | 66 % | 76 % | 74 % |
| Monocytes % | 09% | 10 % | 02 % | 20 % |
| Eosinophils % | 01% | 00 % | 01 % | 01 % |
| Basophils % | 00% | 00 % | 00 % | 01 % |

**HEXANE FRACTION**

| Parameters | Control | Male  1000mg/kg | Female  1000mg/kg | Male  2000mg/kg |
| --- | --- | --- | --- | --- |
| HB | 12.5 gm/dL | 10.5 gm/dL | 8.4 gm/dL | 13.5 gm/dL |
| RBC | 5.9 x 10^12/L | 5.9 x 10^12/L | 4.4 x 10^12/L | 7.3 x 10^12/L |
| HCT | 34% | 37 % | 28 % | 38 % |
| MCV | 57 fL | 62 fL | 65 fL | 52 fL |
| MCH | 21 pg | 18 pg | 19 Pg | 19 Pg |
| MCHC | 37 gm/dL | 28 gm/dL | 30 gm/dL | 36 gm/dL |
| WBC | 3.6 x 10e9/L | 10.1 x 10e9/L | 1.1 x 10e9/L | 5.3 x 10e9/L |
| Platelets | 931 x 10^9/L | 1086 x 10^9/L | 440 x 10^9/L | 1034 x 10^9/L |
| Neutrophils % | 32% | 58 % | 26 % | 31 % |
| Lymphocytes % | 58% | 26 % | 64 % | 60 % |
| Monocytes % | 09% | 16 % | 08 % | 08 % |
| Eosinophils % | 01% | 00 % | 01% | 01 % |
| Basophils % | 00% | 00 % | 01 % | 00 % |

**ETHANOL FRACTION**

| Parameters | Control | Male  1000mg/kg | Female  1000mg/kg | Male  2000mg/kg |
| --- | --- | --- | --- | --- |
| HB | 12.5 gm/dL | 15.5 gm/dL | 10.0 gm/dL | 16.4 gm/dL |
| RBC | 5.9 x 10^12/L | 8.2 x 10^12/L | 5.6 x 10^12/L | 8.6 x 10^12/L |
| HCT | 34% | 53 % | 34 % | 45 % |
| MCV | 57 fL | 64 fL | 60 fL | 52 fL |
| MCH | 21 pg | 19 Pg | 18 Pg | 19 Pg |
| MCHC | 37 gm/dL | 29 gm/dL | 30 gm/dL | 37 gm/dL |
| WBC | 3.6 x 10e9/L | 3.3 x 10e9/L | 3.4 x 10e9/L | 4.1 x 10e9/L |
| Platelets | 931 x 10^9/L | 804 x 10^9/L | 602 x 10^9/L | 859 x 10^9/L |
| Neutrophils % | 32% | 36 % | 22 % | 33 % |
| Lymphocytes % | 58% | 62 % | 75 % | 61 % |
| Monocytes % | 09% | 01 % | 03 % | 06 % |
| Eosinophils % | 01% | 01 % | 00 % | 00 % |
| Basophils % | 00% | 00 % | 00 % | 00 % |

**CHLOROFORM FRACTION**

| Parameters | Control | Male  1000mg/kg | Female  1000mg/kg | Male  2000mg/kg |
| --- | --- | --- | --- | --- |
| HB | 12.5 gm/dL | 9.7 gm/dL | 8.5 gm/dL | 16.3 gm/dL |
| RBC | 5.9 x 10^12/L | 5.3 x 10^12/L | 4.7 x 10^12/L | 8.4 x 10^12/L |
| HCT | 34% | 33 % | 28 % | 46 % |
| MCV | 57 fL | 62 fL | 59 fL | 54 fL |
| MCH | 21 pg | 18 pg | 18 pg | 19 pg |
| MCHC | 37 gm/dL | 29 gm/dL | 31 gm/dL | 36 gm/dL |
| WBC | 3.6 x 10e9/L | 7.1 x 10e9/L | 3.2 x 10e9/L | 8.3 x 10e9/L |
| Platelets | 931 x 10^9/L | 950 x 10^9/L | 507 x 10^9/L | 1077 x 10^9/L |
| Neutrophils % | 32% | 51 % | 13 % | 17 % |
| Lymphocytes % | 58% | 40 % | 84 % | 81 % |
| Monocytes % | 09% | 09 % | 03 % | 00 % |
| Eosinophils % | 01% | 00 % | 00 % | 00 % |
| Basophils % | 00% | 00 % | 00 % | 02 % |

**SERUM UREA AND CREATININE**

**FIXED OIL**

| Parameters | Control | Male  1000mg/kg | Female  1000mg/kg | Male  2000mg/kg |
| --- | --- | --- | --- | --- |
| Creatinine | 0.55mg/dL | 0.46 mg/dL | 0.32 mg/dL | 0.39 mg/dL |
| Urea | 32.1 mg/dL | 40.66 mg/dL | 25.68 mg/dL | 34.24 mg/dL |

**HEXANE FRACTION**

| Parameters | Control | Male  1000mg/kg | Female  1000mg/kg | Male  2000mg/kg |
| --- | --- | --- | --- | --- |
| Creatinine | 0.55mg/dL | 0.33 mg/dL | 0.47 mg/dL | 0.42 mg/dL |
| Urea | 32.1 mg/dL | 29.96 mg/dL | 40.66 mg/dL | 38.52 mg/dL |

**ETHANOL FRACTION**

| Parameters | Control | Male  1000mg/kg | Female  1000mg/kg | Male  2000mg/kg |
| --- | --- | --- | --- | --- |
| Creatinine | 0.55mg/dL | 0.29 mg/dL | No result | 0.40 mg/dL |
| Urea | 32.1 mg/dL | 27.82 mg/dL | No result | 40.66 mg/dL |

**CHLOROFORM FRACTION**

| Parameters | Control | Male  1000mg/kg | Female  1000mg/kg | Male  2000mg/kg |
| --- | --- | --- | --- | --- |
| Creatinine | 0.55mg/dL | No result | No result | 0.39 mg/dL |
| Urea | 32.1 mg/dL | No result | No result | 40.66 mg/dL |
