# Supplementary material for: Antioxidant and anti-inflammatory activities of Centratherum anthelminticum (L.) Kuntze seed oil in diabetic nephropathy via modulation of Nrf-2/HO-1 and NF-κB pathway
Source: BMC Complement Med Ther. 2022 Nov 18;22:301. doi: 10.1186/s12906-022-03776-x (PMC9675141; doi:10.1186/s12906-022-03776-x)
Supplement: Supplementary file 2 — Additional file 2. [file 12906_2022_3776_MOESM2_ESM.docx]

Table 2. Effect of CA FO and its fraction on oral glucose tolerance in rats

| **Groups** | **Time (mints)** | | | |
| --- | --- | --- | --- | --- |
|  | **0** | **30** | **60** | **120** |
| Control | 100.6 ±10.4 | 106 ±10.1 | 111.5 ±20.6 | 86.9 ±11.6 |
| Positive control | 99.4 ±9.8 | 100.6 ±27.9 | 67.1 ±4.3 | 47.3 ±5.7 |
| Negative control | 97.3 ±11.6 | 99.1 ±11.1 | 107 ±10.8 | 83.9 ±7.3 |
| FO (50mg) | 81.1 ±5.7 (-18.9%)^abc^ | 95.4 ±7.1 (-4.6%) | 96.3 ±11.5 (-3.7%)^ab^ | 89.9 ±23 (-10.1%)^b^ |
| FO (100mg) | 75.7 ±5.3 (-24.3%)^bc^ | 92.4 ±5.2 (-7.6%) | 93.7 ±6.5 (-6.3%)^ab^ | 81 ±7.8 (-19%)^b^ |
| FO (200mg) | 104.5 ±9.9 (4.5%) | 111.8 ±16.3 (11.8%) | 90.5 ±12.7 (-9.5%)^ab^ | 84.3 ±11.3 (-15.7%)^b^ |
| FO (400mg) | 80.5 ±9.6 (-19.5%)^abc^ | 93.9 ±8.2 (-6.1%) | 108.3 ±12.9 (8.3%)^b^ | 91.8 ±7.3 (-8.2%)^b^ |
| FO (600mg) | 81.1 ±10.4 (-18.9%)^abc^ | 102.9 ±11.1 (2.9%) | 101 ±21 (1%)^b^ | 83.6 ±12.9 (-16.4%)^b^ |
| HF (50mg) | 80.4 ±6.8 (-19.6%)^abc^ | 95.3 ±9.7 (-4.7%) | 97.3 ±10.5 (-2.7%)^ab^ | 90.9 ±12.4 (-9.1%)^b^ |
| HF (100mg) | 79.3 ±6.1 (-20.7%)^abc^ | 95.7 ±7.8 (-4.3%) | 96.9 ±22.6 (-3.1%)^b^ | 98.1 ±7.6 (-1.9%)^bc^ |
| HF (200mg) | 93.9 ±11 (-6.1%) | 103.7 ±9.6 (3.7%) | 98.8 ±15.8 (-1.2%)^b^ | 83.6 ±12.5 (-16.4%)^b^ |
| HF (400mg) | 79.1 ±9.7 (-20.9%)^abc^ | 102.6 ±8.6 (2.6%) | 111.6 ±10.4 (11.6%)^b^ | 92 ±7.4 (-8%)^b^ |
| HF (600mg) | 74.4 ±6.1 (-25.6%)^abc^ | 105.1 ±22.7 (5.1%) | 86.4 ±23.1 (-13.6%)^abc^ | 81.9 ±15.5 (-18.1%)^b^ |
| CF (50mg) | 71.9 ±6.5 (-28.1%)^abc^ | 86.1 ±6.5 (-13.9%)^ab^ | 98.6 ±4.9 (-1.4%)^b^ | 85 ±8.2 (-15%)^b^ |
| CF (100mg) | 80.7 ±3.7 (-19.3%)^abc^ | 101.1 ±21.8 (1.1%) | 106.6 ±11.7 (6.6%)^b^ | 104.3 ±16.8 (4.3%)^abc^ |
| CF (200mg) | 102.5 ±8.8 (2.5%) | 105.9 ±22.5 (5.9%) | 107.1 ±15.4 (7.1%)^b^ | 96.8 ±6.4 (-3.2%)^bc^ |
| CF (400mg) | 79.9 ±7 (-20.1%)^abc^ | 111.3 ±17 (11.3%) | 107.8 ±7.3 (7.8%)^b^ | 101.6 ±12.7 (1.6%)^abc^ |
| CF (600mg) | 78.8 ±6.5 (-21.2%)^abc^ | 109.8 ±23.2 (9.8%) | 85.1 ±6.7 (-14.9%)^abc^ | 111 ±6.7 (11%)^bc^ |
| EF (50mg) | 78.1 ±9.3 (-21.9%)^abc^ | 92 ±9.4 (-8%) | 90.4 ±18.4 (-9.6%)^abc^ | 76.3 ±15.3 (-23.7%)^b^ |
| EF (100mg) | 80.9 ±8.6 (-19.1%)^abc^ | 98.3 ±11 (-1.7%) | 99 ±13.1 (-1%)^b^ | 88.8 ±16.3 (-11.2%)^b^ |
| EF (200mg) | 98.4 ±9.7 (-1.6%) | 101.5 ±14.1 (1.5%) | 99.9 ±11.2 (-0.1%)^b^ | 80.4 ±12 (-19.6%)^b^ |
| EF (400mg) | 78.8 ±6.5 (-21.2%)^abc^ | 102.4 ±11.9 (2.4%) | 108.6 ±9.8 (8.6%)^b^ | 103.6 ±7.9 (3.6%)^abc^ |
| EF (600mg) | 76.8 ±7.4 (-23.2%)^abc^ | 96.1 ±18.2 (-3.9%) | 99.4 ±20.3 (-0.6%)^b^ | 97.5 ±7.3 (-2.5%)^abc^ |

All values are expressed as mean ± SEM (n=12)

Value in parenthesis shows percent glycemic change (+) increase/ (-) decrease

^a^ p value <0.05 as compared to control

^b^ p value <0.05 as compared to positive control

^c^ p value <0.05 as compared to negative control
